# Comparison between Vascular Endothelial Growth Factor and Platelet-Derived Growth Factor Levels in Rhegmatogenous Retinal Detachment

**DOI:** 10.1155/2021/2688837

**Published:** 2021-12-06

**Authors:** Andi Muhammad Ichsan, Dyah Ayu Windy, Habibah Setyawati Muhiddin, Budu, Muhammad Nasrum Massi, Itzar Chaidir Islam

**Affiliations:** ^1^Department of Ophthalmology, Medical Faculty, Hasanuddin University, Makassar, Indonesia; ^2^Department of Microbiology, Medical Faculty, Hasanuddin University, Makassar, Indonesia

## Abstract

**Introduction:**

This study aimed to assess vascular endothelial growth factor (VEGF) and platelet-derived growth factor (PDGF) levels within vitreous and blood serum samples of patients with rhegmatogenous retinal detachment (RRD) and their relationship to the development of proliferative vitreoretinopathy (PVR).

**Methods:**

Seventeen eyes of patients with RRD were included in the RRD group and divided into three subgroups: RRD without PVR, RRD with PVR grades A and B, and RRD with PVR grade C. Five control eyes (nucleus and intraocular lens drop) were included in this study. Blood serum and vitreous samples were collected during vitrectomy. VEGF-A and PDGF-AA levels were determined by enzyme-linked immunosorbent assay.

**Results:**

The mean vitreous VEGF-A level in the RRD group was 131.71 ± 58.25 pg/mL, and the mean vitreous PDGF-AA level was 174.62 ± 65.17 pg/mL. Both levels were significantly higher in the RRD group compared with the control group (*p* < 0.05). Vitreous VEGF-A and PDGF-AA levels were the highest in RRD with PVR grade C subgroup, with mean levels of 179.87 ± 21.02 pg/mL and 229.44 ± 14.09 pg/mL, respectively (*p* < 0.05). The vitreous VEGF-A/PDGF-AA ratios in the RRD subgroups were completely different.

**Conclusion:**

Based on the tendency of VEGF-A and PDGF-AA levels, RRD surgery has to be performed as soon as possible prior to retinal cell death and membrane proliferative formation.

## 1. Introduction

Rhegmatogenous retinal detachment (RRD) induces ischemic conditions of photoreceptor, which triggers the expression of many growth factors and cytokines, such as vascular endothelial growth factor (VEGF), platelet-derived growth factor (PDGF), hepatocyte growth factor, epidermal growth factor, transforming growth factors *α* and *β*, granulocyte-colony stimulating factor, fibroblast growth factors, insulin-like growth factor-I, cytokines (interleukin- (IL-) 1, IL-6, IL-8, IL-10, and interferon *γ*), matrix metalloproteinase, and chemokine in the vitreous and subretinal fluid [1–3].

VEGF is an angiogenic and vasopermeable factor specific to endothelial cells that are produced by various cells, such as retinal pigment epithelium (RPE), ganglion cells, glial cells, smooth and pericidal muscle cells in the vessel wall, and choroidal fibroblasts. VEGF can be regulated by hypoxic conditions [4]. The neurosensory layer of the retina is separated from the retinal pigment epithelial layer in a patient with RRD and induces hypoxic conditions in the outer nucleus caused by separation of the outer nucleus cells from their oxygen supply from the choroid. The outer nuclear layer of the retina in patients with RRD also shows upregulation of VEGF [5].

Another growth factor that increased after RRD is PDGF, a growth factor that originates from thrombocyte and can induce proliferation and migration of various types of cells, including endothelial cells, smooth muscle cells, and mesenchymal cells. After retinal detachment, the production of PDGF-A by RPE cells increases, and the total amount of PDGF-AA in the eye can increase to a level sufficient for the formation of epiretinal membranes, which can cause traction [6, 7].

The high incidence of proliferative vitreoretinopathy (PVR), which is one of the most common complications of RRD, seemed to have a relationship with increased VEGF and PDGF levels in the vitreous and subretinal fluid after RRD [8–10]. Based on clinical experience, surgical treatment in RRD cases with PVR no longer provides significant visual outcomes. This study aimed to assess the VEGF and PDGF levels within vitreous and blood serum samples of patients with RRD and their relationship to the development of PVR.

## 2. Materials and Methods

### 2.1. Study Design and Subjects

This study is an analytical observational investigation using cross-sectional design. We registered consecutively attending adults (age >18 years) who were clinically affirmed to have RRD, nucleus drop, and intraocular lens (IOL) drop. The inclusion criterion for the RRD group was pars plana vitrectomy. The RRD group was divided into three subgroups: subgroup I, RRD without PVR; subgroup II, RRD with PVR grades A and B; and subgroup III, RRD with PVR grade C (updated Retina Society Classification for PVR) [11]. The control group comprised of patients who underwent pars plana vitrectomy for complete nucleus drop or IOL drop. Previous intraocular medical procedure in the last two years, postocular inflammation, rubeosis iridis or neovascular glaucoma, and diabetic retinopathy were the exclusion criteria for the RRD group.

This investigation was conducted in September 2018 to March 2019 and approved by the Institutional Review Board of the Faculty of Medicine, Hasanuddin University of Makassar. All subjects provided written informed consent for all medical procedures, such as pars plana vitrectomy, and underwent blood serum and vitreous sampling as per the principles of the Declaration of Helsinki.

### 2.2. Sample Collection and Measurement of VEGF-A and PDGF-AA Levels

Blood tests were collected from all subjects during pars plana vitrectomy and left for 2 h at room temperature until coagulation. Serum was isolated by centrifugation at 4°C for 15 min at 1000 ×g, separated into aliquots, and then stored at −80°C until measurement.

At the beginning of vitrectomy, 0.5–0.8 mL of undiluted vitreous sample was collected by aspiration into a 1 mL sterile needle appended to the vitreous cutter using the Constellation® Vision System (Alcon Laboratories, Fort Worth, TX, USA) with the stopcock of the infusion shut. The sample was moved into sanitized Corning microcentrifuge tubes (1.5 mL), put promptly on ice, and centrifuged for 20 min at 4°C at 1000 g. Supernatant without residue was isolated into aliquots and quickly frozen at −80°C until measurement.

Elabscience® Human VEGF-A and PDGF-AA ELISA Kit for enzyme-linked immunosorbent assay (ELISA) examination were used to measure vitreous and blood serum VEGF-A and PDGF-AA levels. Each test was conducted in duplicate as indicated by the manufacturer's directions, utilizing 100 *μ*L aliquots of vitreous and blood serum samples, which are diluted as needed to conform to the detection range of the measurement, and finally contain a similar measure of protein. The data are presented per milligram of protein. The optical thickness was set at 450 and 570 nm, utilizing an absorption spectrophotometer.

### 2.3. Statistical Analyses

All levels are shown as mean ± standard deviation (SD), and all data are provided in the table and figures. Statistical analysis was performed using Statistical Package for the Social Sciences version 22.0. The contrasts between groups were calculated utilizing independent *t* test and one-way ANOVA. A *p* value < 0.05 was considered statistically significant.

### 2.4. Ethical Statement

This study protocol was reviewed and approved by The Ethics Committee of Medical Research, Faculty of Medicine, Hasanuddin University in accordance to the Declaration of Helsinki with approval number: 1111/H4.8.4.5.31/PP36-KOMETIK/2018. Written informed consent was obtained from the patient for medical examination, any accompanying images, and study publication.

## 3. Results

### 3.1. Patients' Characteristics

A total of 22 eyes were selected, consisting of 17 eyes of 17 patients with RRD and five eyes of five patients with nucleus and IOL drop as controls. Thirteen eyes had best-corrected visual acuity categorized as blind, two eyes as severe visual impairment, and two eyes as moderate visual impairment (Table 1).

### 3.2. VEGF-A and PDGF-AA Levels in Patients with RRD and Controls

The mean vitreous VEGF-A levels in the RRD group (131.71 ± 58.25 pg/mL) were higher compared wih that in the control group (88.47 ± 56.95 pg/mL) (*p* < 0.05). The mean vitreous PDGF-AA levels in the RRD group (174.62 ± 65.17 pg/mL) was also higher compared with that in the control group (33.15 ± 19.51 pg/mL) (*p* < 0.05) (Table 2). The blood serum levels of both VEGF-A and PDGF-AA in the RRD group (706.20 ± 185.29 pg/mL and 751.58 ± 129.27 pg/mL, resp.) were not significantly different from that of the control group (674.34 ± 360.12 pg/mL and 657.72 ± 840.54 pg/mL, resp.) (*p* ≥ 0.05) (Table 2). Interestingly, serum VEGF-A and PDGF-AA levels were higher compared with the vitreous levels in both the RRD and control groups.

### 3.3. VEGF-A and PDGF-AA Levels in Patients with RRD Based on Duration, Risk Factors, Tear Type, and Area of RRD

Based on the duration of RRD, there were nine patients (52.9%) with an onset of less than one month, four patients (23.5%) with an onset of one month to one year, and four patients (23.5%) with an onset of more than one year. The risk factors for RRD were high myopia in four patients (23.5%), trauma in three patients (17.6%), and idiopathic condition in 10 patients (58.8%). Retinal tears were divided into two types: flap/horseshoe tear in 13 patients (76.5%) and giant tear in four patients (23.5%). Based on the area of RRD, there were nine patients (52.9%) with two-quadrant RRD, five patients (29.4%) with three-quadrant RRD, and three patients (17.6%) with four-quadrant RRD. Based on its duration, there was a significant difference in vitreous VEGF-A and PDGF-AA levels in the RRD group (*p* < 0.05) (Table 3). The levels of these two growth factors in the vitreous of patients with RRD increase in line with the increasing duration of retinal detachment. Meanwhile, no significant differences were found in the serum VEGF-A and PDGF-AA levels of patients with RRD based on their duration (*p* ≥ 0.05).

In the risk factor group, there were significant differences in the vitreous PDGF-AA levels in the high myopia group, trauma group, and idiopathic group (120.97 ± 64.50 pg/mL, 241.54 ± 14.68 pg/mL, and 176.00 ± 57.79 pg/mL, resp.) (*p* < 0.05). The highest vitreous PDGF-AA level was found in the trauma group. However, the serum VEGF-A and PDGF-AA levels in the risk factor group were not statistically significantly different (*p* ≥ 0.05).

There were no critical contrasts in the vitreous and blood serum levels of both VEGF-A and PDGF-AA in the tear type and RRD region groups (*p* ≥ 0.05).

### 3.4. VEGF-A and PDGF-AA Levels in Patients with RRD Based on the Grading of PVR

The RRD group was divided into three subgroups according to the updated Retina Society Classification of PVR [11]. In light of the RRD subgroups, subgroup I (no PVR) was noted in four patients, while subgroup II (PVR grades A and B) was noted in four patients and subgroup III (PVR grade C) in nine patients.

There was critical contrast in both vitreous VEGF-A and PDGF-AA levels among all RRD subgroups (*p* < 0.05) (Table 4). There was an intriguing scheme of VEGF-A levels in the vitreous sample among the RRD subgroups, where the vitreous VEGF-A level was lowest in subgroup II and increased in subgroup III, although there was no huge distinction in the blood serum levels of both VEGF-A and PDGF-AA among all RRD subgroups (*p* ≥ 0.05).

There was a significant difference in the vitreous VEGF-A/PDGF-AA ratio among the RRD subgroup (*p* < 0.05). There were interesting changes in the ratio of vitreous VEGF-A/PDGF-AA ratio among these subgroups, where, in subgroup I, the vitreous VEGF-A levels were higher compared with PDGF-AA vitreous levels. In contrast, in subgroup II, the vitreous PDGF-AA levels were higher compared with VEGF-A vitreous levels, whereas, in subgroup III, both vitreous VEGF-A and PGDF-AA levels were increased almost equally (Table 5). There was no critical distinction in the serum VEGF-A/PDGF-AA ratio among all RRD subgroups.

## 4. Discussion/Conclusion

Retinal ischemia in RRD is caused by the separation of the retinal neurosensory layer from its oxygen supply from the choroid. If this condition is not immediately corrected, it will cause damage and death of retinal cells [5]. Based on clinical experience, surgical treatment in late RRD cases no longer provides a significant visual outcome.

This study was conducted to evaluate the VEGF-A and PDGF-AA levels in the vitreous and blood serum in RRD cases. We suggest that, in conditions where VEGF-A level increases, retinal cells are in the hypoxic stage, and if this condition continues, the retinal cells will no longer be viable. At the time when the retinal cells are no longer viable, the VEGF-A level will decrease. In addition to assessing VEGF-A level, we also determined factors that might cause the emergence of PVR, which aggravates RRD conditions. Therefore, we also assessed the PDGF-AA level, which plays an important role in the formation of proliferative membrane in RRD cases.

In this study, we found a significant increase in vitreous VEGF-A levels in the RRD group rather than in the control group. A study with the same results was also suggested by Rasier et al. [4], which found a significant increase in VEGF levels in patients with vitreous RRD compared with non-RRD groups. Moreover, Su et al. [5] and Yalcinbayir et al. [11] conducted a study that assessed VEGF levels in the subretinal fluid in patients with RRD and also found a significant increase in VEGF levels in these patients compared with those in controls. It is known that VEGF levels increase in various conditions, such as hypoxia and intraocular inflammation [4]. Various types of cells in the eye, such as RPE, ganglion cells, glia cells, smooth and pericyte muscle cells in the vessel wall, and choroidal fibroblasts, express mRNA for VEGF production [5]. In the case of RRD, VEGF levels increase due to damage to the blood retina barrier (BRB) after RRD. Su suggested that when the sensory layer of the retina detaches from the RPE layer, an increase in the external nuclear layer leads to a hypoxic state in the outer nuclear layer that is separated from its oxygen supply from the choroid [5].

In our study, we found significant differences in vitreous VEGF-A levels based on the duration (<1 month, 1 month to 1 year, and >1 year), where the vitreous VEGF-A levels increase in line with the duration of RRD. Su et al. also suggested that there was a significant correlation between VEGF level and duration of RRD where there was an increase in VEGF levels along with the duration of RRD [5]. Yalcinbayir et al. suggested that, in chronic or inadequately treated RRD cases, neovascularization of the iris may occur due to the increase in VEGF levels along the course of the disease, similar to the parabolic slope where the rising side is noted in the chronic phase of RRD [12].

In addition to the significant increase in vitreous VEGF-A levels, this study also obtained significant difference in vitreous PDGF-AA levels in RRD cases compared with controls. A similar result was found in the study conducted by Mori et al. which showed an increase in PDGF-AA level in experimental mice with retinal detachment [13]. PDGF is a mitogen and chemoattractant for RPE cells, retinal glial cells, and pericidal cells (cells involved in the formation of aneurysms due to weakness and discontinuity of the capillary blood vessel walls), where the expression in the eye increases after RD [6, 7, 13–15]. Campochiaro et al. suggest that, after RRD, the production of PDGF-A by RPE cells increases and PDGF-A is only specifically bound to the PDGF*α* receptor, which is present in the retinal glial cells and RPE so that the total PDGF-AA in the eye will increase to the appropriate level to cause proliferative membrane formation [6].

While serum VEGF-A and PDGF-AA levels in our study were not significantly different compared with those in the controls, the levels were equally increased compared with vitreous VEGF-A and PDGF-A levels in both the RRD and control groups. Since RRD is a local condition of the eye so that the vitreous VEGF-A and PDGF-AA levels are significantly different from those in the control group, the difference in serum VEGF-A and PDGF-AA levels is largely influenced by systemic factors that represent all organs so that the value is not significantly different between the RRD and control groups.

PVR is a complication from RRD that can cause a case of redetachment. PVR is formed after the RRD event and is characterized by the growth and contraction of cellular membranes in the vitreous cavity and on the inner surface of the retina [1, 16]. This PVR is formed by a tear process in the retina that results in the exposure of cells to growth factors and cytokines present in the vitreous. This causes the cellular response in the form of migration of RPE and glial cells to the vitreous, where these cells will proliferate, synthesize the matrix of extracellular proteins, form a membrane, and contract, which will pull the attached retina that has been treated before or make new tears [1, 16–19]. In this study, we divided the RRD group into three subgroups: RRD without PVR, RRD with PVR grades A and B, and RRD with PVR grade C. We assessed the vitreous and serum VEGF-A and PDGF-AA levels based on this grading of PVR.

There was a significant difference in the vitreous VEGF-A levels among the three RRD subgroups. These results are consistent with those of the study by Ricker et al. which found an increase in VEGF-A vitreous levels up to two to three times in the PVR group compared with the RRD without PVR group [20]. Because PVR is characterized by an avascular membrane, it is concluded that VEGF has a function other than inducing angiogenesis. Chen et al. [16] hypothesized that there was development of VEGF interactions with nonendothelial cells in the eye, such as RPE, photoreceptor cells, and Mϋller cells. Moreover, VEGF also induces monocyte activation that is manifested from induction of monocyte chemotaxis. This shows the possibility of VEGF having pleiotropic effects on various cell types, such as RPE and monocyte cells, which have an important role in PVR formation [21].

We also found a significant difference in the vitreous PDGF-AA level among the three RRD subgroups, with the highest PDGF-AA level found in subgroup III (RRD with PVR grade C). This finding was in line with the result of study conducted by Cui et al. [8] They found that PDGF in the vitreous was highly correlated with PVR, where eight of nine patients with PVR have detected PDGF level in their vitreous, while only one of six patients with other retinal diseases required surgical treatment with detected PDGF level in their vitreous. Their study showed that cells that express PDGF*α* receptors induce PVR more effectively compared with cells that express PDGF*β* receptors. When there is a retinal tear, RPE cells and other cells, such as platelets, fibroblasts, and smooth muscle cells, increase the expression of PDGF-A, which are bound to PDGF*α* receptor in RPE and glial cells. This binding induces the process of the RPE and glial cell migration to vitreous and then proliferates and synthesizes the matrix of extracellular proteins to form a proliferative membrane as a wound healing response. The worse the grading of PVR, the more the PDGF-A bonds to PDGF*α* receptors, so it will cause the higher PDGF-AA level found in the vitreous [8, 13].

In this study, we found the differences in the ratio of vitreous VEGF-A/PDGF-AA ratio among all RRD subgroups based on the grading of PVR, where, in subgroup I (RRD group without the PVR), vitreous VEGF-A/PDGF-AA ratio showed slightly higher vitreous VEGF-A levels than vitreous PDGF-AA levels with a ratio of 1.2 : 1. We suggest that, in the early phase of RRD, VEGF-A and PDGF-AA receptors were activated by exposure to growth factors from the vitreous that enter the subretinal space so that the levels of both growth factors are increased [13]. However, the VEGF-A level in the early phase of RRD was higher than the PDGF-AA level. This is in accordance with our suggestion that, in this phase, photoreceptor cells and retinal cells in the outer nuclear and plexiform layers have hypoxic conditions that trigger a response to increased rates of VEGF-A levels. Whickam et al. suggested that the hypoxic condition of the retina inactivates the prolyl hydroxylase, causing hypoxia-inducible factor-1*α* (HIF-1*α*) to accumulate and bind to HIF-1*β* to form HIF-1, which translocates to the nucleus and binds to the hypoxia response element located in the promoter region of VEGF gene and other genes [22]. This theory explains how the hypoxic retinal cells can increase the VEGF-A level. In this phase, retinal cells are still viable, even though they have hypoxic condition. This is the most appropriate time for immediate surgery to reattach the neurosensory layer to the RPE and choroid.

In an experimental study of retinal detachment, it was found that, within 12 h, the outer photoreceptor segment showed structural damage. Initially, the distal part of the outer segment turned into a vacuole, and within 24–72 h, the entire outer segment of rod and cone cells became shorter and had significant damage with discus damage [23, 24]. Therefore, in the case of RRD, surgical treatment should be performed within <24 h of onset, before the occurrence of structural damage, so that recovery of the retinal cells can be optimal.

The vitreous VEGF-A/PDGF-AA ratio in subgroup II (RRD group with PVR grades A and B) was 3 : 10, where PDGF-AA levels were higher than VEGF-A levels. We suggested that it is due to the cellular response to the healing process in the form of cell migration, proliferation, survival, and production of cellular extracellular matrix proteins, where PDGF-AA is a growth factor that plays a major role in this process compared with VEGF-A. In this phase, the process of fibrosis tissue formation occurs rapidly. The study conducted by Mori et al. showed that, after retinal detachment, the production of PDGF-A by RPE cells increased, and the total amount of PDGF-AA in the eye could increase at the appropriate level to cause proliferative membrane formation [12]. At this condition, VEGF-A levels have decreased because photoreceptor and retinal cells are no longer viable so that the oxygen demand decreased and did not provide a response for VEGF-A production.

In subgroup III (RRD group with PVR grade C), it was found that the vitreous VEGF-A/PDGF-AA ratio increased to 7 : 10, whereas both vitreous VEGF-A and PDGF-AA levels increased to almost the same level. The hypothesis of an increase in the levels of these two growth factors is due to the chronicity of the RRD itself and the damage to the old BRB. Yu et al. [25] obtained a number of significant new proteins in the patients with vitreous PVR patients, in which these proteins play a role in the inflammatory process and adhesion cells through the coagulation cascade process and complement. This coagulation cascade process and complement causes cell lysis, cell adhesion, migration, proliferation, fibrin degradation, and inflammation, which lead to chronic damage of BRB. This chronic BRB damage is suggested to be related to the activation of VEGF-A formation from the choroid in severe PVR conditions. In addition to chronic BRB damage, we also suggest an increase in VEGF-A level in this phase as a result of an increase in VEGF165b isoform, which is one of the anti-angiogenic VEGF-A isoforms. Ricker et al. found that the levels of VEGF165b isoforms were as much as 65% of the total VEGF in patients with vitreous PVR and macular holes, which explain a lack of blood vessel in PVR membranes [3]. We suggested that the increase in VEGF-A isoform levels also leads to an increase in the total VEGF-A levels in RRD with PVR grade C conditions. Moreover, a study by Pennock and Kazlauskas [26] and Lei et al. [27] suggested that high PDGF levels can trigger activation of PDGF*α* receptors through non-PDGF indirect pathways, where VEGF-A competes with PDGF in activating PDGF*α* receptors [26, 27]. Presumably, in the case of severe PVR, where PDGF levels significantly increased, they could trigger non-PDGF activity. VEGF levels also increase to compete with PDGF and facilitate non-PDGF indirect pathways in activating PDGF*α*. Therefore, in this condition, the VEGF-A/PDGF-AA ratio should be equal in the patient's vitreous.

As a conclusion, during the acute phase of RRD, vitreous VEGF-A levels significantly increase due to hypoxic conditions of photoreceptor and retinal cells. This phase is the right time to perform surgery to reattach the living photoreceptor and retinal cells to the RPE so that these cells regain oxygen and nutrients from the choroid and can function properly again. Meanwhile, in the chronic phase of RRD, which is accompanied by the formation of PVR, photoreceptor cells and retinal cells were inviable. Further reattachment surgery will no longer provide meaningful visual outcome. As RRD is only a local process, these two growth factors are not suitable for serum biomarkers. Further investigations and experimental studies using animal models may be required in the future.

## Figures and Tables

**Table 1:** Patients' characteristics.

Variable	Value	Groups		(%)
		RRD	Control	
*Sex*				
Male	13	9	4	59.1
Female	9	8	1	40.9
*Age*				
<50 years	13	11	2	59.1
≥50 years	9	6	3	40.9
*Side of the eye*				
OD	8	7	1	36.4
OS	14	10	4	63.6
*BCVA*				
Moderate visual impairment	7	2	5	31.8
Severe visual impairment	2	2	0	9.1
Blind	13	13	0	59.1

The data are expressed as number (according to data variable). OD oculus dextra; OS oculus sinistra; BCVA; best corrected visual acuity.

**Table 2:** VEGF-A and PDGF-AA levels.

Variable	Group	Level (pg/ml)	*p*
VEGF-A vitreous	RRD	131.71 ± 58.25	0.009^*∗*^
	Control	88.47 ± 56.95	

VEGF-A serum	RRD	706.20 ± 185.29	0.789
	Control	674.34 ± 360.12	

PDGF-AA vitreous	RRD	174.62 ± 65.17	0.0001^*∗*^
	Control	33.15 ± 19.51	

PDGF-AA serum	RRD	751.58 ± 129.27	0.644
	Control	657.72 ± 840.54	

The data are expressed as mean ± SD. ^*∗*^Significant difference between RRD and control group using independent *T* test (*p* < 0.05). RRD: rhegmatogenous retinal detachment; VEGF-A: vascular endothelial growth factor-A; PDGF-AA: platelet derived growth factor-AA.

**Table 3:** VEGF-A and PDGF-AA levels based on the duration of disease.

Variable	Groups	*n*	Level (pg/ml)	*p*
VEGF-A vitreous	<1 month	9	97.29 ± 42.50	0.012^*∗*^
	1 month–1 year	4	151.44 ± 65.46	
	>1 year	4	189.46 ± 21.85	

VEGF-A serum	<1 month	9	696.18 ± 183.13	0.521
	1 month–1 year	4	794.10 ± 255.26	
	>1 year	4	640.88 ± 110.60	

PDGF-AA vitreous	<1 month	9	129.46 ± 54.05	0.002^*∗*^
	1 month–1 year	4	212.01 ± 34.67	
	>1 year	4	238.83 ± 13.28	

PDGF-AA serum	<1 month	9	706.69 ± 92.79	0.335
	1 month–1 year	4	798.79 ± 200.66	
	>1 year	4	805.38 ± 114.08	

The data are expressed as mean ± SD. ^*∗*^Significant difference among RRD group using one way ANOVA test (*p* < 0.05).VEGF-A: vascular endothelial growth factor-A; PDGF-AA: platelet derived growth factor-AA.

**Table 4:** VEGF-A and PDGF-AA levels in each RRD subgroup.

Variable	Groups	*n*	Level (pg/ml)	*p*
VEGF-A vitreous	I	4	104.69 ± 11.44	0.0001^*∗*^
	II	4	50.38 ± 6.02	
	III	9	179.87 ± 21.02	

VEGF-A serum	I	4	670.16 ± 274.56	0.455
	II	4	811.90 ± 81.49	
	III	9	675.25 ± 174.91	

PDGF-AA vitreous	I	4	81.83 ± 9.35	0.0001^*∗*^
	II	4	144.06 ± 17.07	
	III	9	229.44 ± 14.09	

PDGF-AA serum	I	4	706.43 ± 26.62	0.679
	II	4	790.77 ± 38.56	
	III	9	754.23 ± 175.50	

Group classifications: I: RRD with no PVR; II: RRD with PVR grade A and B; III: RRD with PVR grade C. ^*∗*^Significant difference using one way ANOVA test among all RRD subgroups (*p* < 0.05). RRD: rhegmatogenous retinal detachment; VEGF-A: vascular endothelial growth factor-A; PDGF-AA: platelet derived growth factor-AA; PVR: proliferative vitreoretinopathy.

**Table 5:** VEGF-A and PDGF-AA level ratio in each RRD subgroup.

Variable	Groups	*n*	Level (pg/ml)	*p*
VEGF-A/PDGF-AA vitreous	I	4	1.29 ± 0.19	0.0001^*∗*^
	II	4	0.35 ± 0.03	
	III	9	0.78 ± 0.06	

VEGF-A/PDGF-AA serum	I	4	0.95 ± 0.39	0.943
	II	4	1.03 ± 0.13	
	III	9	0.96 ± 0.43	

Group classifications: I: RRD with no PVR; II: RRD with PVR grade A and B; III: RRD with PVR grade C. ^*∗*^Significant difference using one way ANOVA test among all RRD subgroups (*p* < 0.05). RRD: rhegmatogenous retinal detachment; VEGF-A: vascular endothelial growth factor-A; PDGF-AA: platelet derived growth factor-AA; PVR: proliferative vitreoretinopathy.

## Data Availability

The data that support the findings of this study are available from the corresponding author upon reasonable request.
